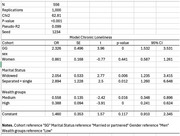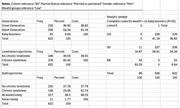# Generational differences in chronic loneliness reported by people with dementia over 14 years: results from the English Longitudinal Study of Ageing

**DOI:** 10.1002/alz70858_102999

**Published:** 2025-12-25

**Authors:** Thamara Tapia‐Muñoz, Claudia Miranda‐Castillo

**Affiliations:** ^1^ Millennium Institute for Care Research, Santiago, Chile; ^2^ Universidad Andres Bello, Santiago, Santiago, Chile; ^3^ University College London, London, United Kingdom; ^4^ Universidad Andres Bello, Santiago, Chile; ^5^ Millenium Institute for Research in Depression and Personality, Santiago, Chile

## Abstract

**Background:**

There is a need to better understand loneliness trajectories in people with dementia and how these may vary across generations.

**Method:**

We conducted a secondary analysis of the English Longitudinal Study of Ageing (ELSA). Across nine waves (2002–2019), 622 participants reported having a diagnosis of dementia. We identified those who reported chronic loneliness at any point, defined as a score of ≥6 on the three‐item R‐UCLA scale for at least three consecutive waves. Generations were categorised by birth year: the Greatest Generation (GG, born up to 1925; 36.82%), the Silent Generation (SG, 1926–1945; 54.34%), and the Baby Boomers (BB, 1946–1964; 8.84%). Due to sample size constraints, only GG and SG were compared (*N* = 556). Loneliness was measured from wave 2 (2005). Logistic regression models were used, with bootstrap errors based on 1,000 iterations.

**Result:**

Between 2005 and 2019, 376 participants with dementia (67%) reported chronic loneliness. Logistic regression indicated that the likelihood of reporting chronic loneliness was associated with birth cohort, marital status, and wealth level (in tertiles). Individuals with dementia from the GG were more likely to report chronic loneliness (OR = 2.32; 95% CI: 1.53–3.53). No significant differences were found in marital status or wealth level between the two generations. No gender differences were observed.

**Conclusion:**

Previous research has noted higher loneliness in earlier‐born cohorts, and people with dementia appear to be no exception. Further investigation is required to explore the factors underpinning these generational differences in chronic loneliness among individuals with dementia.